# Socioeconomic Risk and School Readiness: Longitudinal Mediation Through Children's Social Competence and Executive Function

**DOI:** 10.3389/fpsyg.2018.01544

**Published:** 2018-08-28

**Authors:** Rosemarie E. Perry, Stephen H. Braren, Clancy Blair, The Family Life Project Key Investigators, Lynne Vernon-Feagans

**Affiliations:** Neuroscience and Education Laboratory, Department of Applied Psychology, Institute of Human Development and Social Change, New York University, New York, NY, United States

**Keywords:** executive function, social competence, early-life adversity, poverty, social skills, social behavior, development, longitudinal

## Abstract

The association of socioeconomic status with academic readiness and school achievement is well established. However, the specific contributions of cognitive and social aspects of self-regulation, and potential reciprocal relations between them in the prediction of school readiness and early school achievement have not previously been examined. This study examined mediational processes involving children's executive function (EF) skills at 58 months and Grade 1 (G1) and social competence in Kindergarten (K) and G1, as potential pathways by which early-life poverty-related risks influence Grade 2 (G2) math and reading achievement. Data came from the Family Life Project, which is a prospective longitudinal study of 1,292 children and families followed from birth in primarily low-income, non-urban counties in Pennsylvania (PA) and North Carolina (NC). Autoregressive cross-lagged mediation analyses indicated that EF at 58 months through EF at G1 mediated negative associations between cumulative risk exposure and academic skills, with this pathway mediating 36% of the total effect. Furthermore, social competence at K through EF at G1 mediated negative associations between early-life cumulative socioeconomic risk and academic skills, mediating 16% of the total effect. These findings provide evidence that poverty-related risks can influence school readiness and academic achievement via EF. Additionally, these results provide preliminary support for the premise that social competence through EF is a pathway by which cumulative poverty-related risk predicts early academic competence. Our findings are consistent with studies demonstrating developmental associations between EF and social competence. Furthermore, our findings are consistent with prekindergarten programs for children in poverty that emphasize both cognitive and social aspects of self-regulation.

## Introduction

Decades of research have converged on the finding that growing up in poverty can negatively impact a child's academic abilities and achievement throughout the lifespan (Lacour and Tissington, 2011; Solano and Weyer, 2017). Such socioeconomic disparities are globally observed and emerge early in life, with exposure to poverty increasing the probability that a child will enter school behind their more advantaged peers in emergent math and literacy skills (Ryan et al., 2006; Engle and Black, 2008). Longitudinal data suggest that early gaps in measures of academic readiness for socioeconomically underprivileged children persevere and amplify as children progress through school, contributing to the emergence of inequalities in health care, employment opportunities, and judicial involvement (Huffman et al., 2001). Furthermore, prior research has demonstrated that exposure to multiple poverty-related risks are related to poorer child outcomes above and beyond the effects of any single factor (Evans et al., 2013). Thus, isolating early factors that might influence children's subsequent achievement has become of great importance for identifying children at high risk of early school failure, as well as the design of targeted preventative interventions.

Acknowledged factors by which to promote school readiness and later achievement include instructional strategies for building emergent literacy (letter recognition, phonemic sensitivity) and numeracy skills (counting) (Wasik et al., 2006; Howes et al., 2008). However, core cognitive and social-emotional skills have been demonstrated to be equally important facets of school readiness (Campbell and Stauffenberg, 2008; Blair and Raver, 2015; Eickmann et al., 2016). These broader competencies promote “readiness to learn,” an essential precursor to successful content-based learning (Campbell and Stauffenberg, 2008; Blair and Raver, 2015; Eickmann et al., 2016). For example, throughout the preschool years, development of inhibitory control and prosocial behaviors fosters the ability to sit still, as well as listen and attend to instructions and rules (McClelland et al., 2006). Furthermore, children with high levels of cognitive control and social-emotional skills are more able to attend to academic tasks, follow teachers' instructions, plan, exchange knowledge with peers, model appropriate peer behavior, and devote resources to learning, relative to their less competent peers (Denham et al., 2013).

As it has become evident that core cognitive and social-emotional competencies are central to school readiness, it has been proposed that children living in poverty face a multitude of risks which threaten the acquisition of these core competencies (Kaiser et al., 2000; Blair and Razza, 2007; Hughes et al., 2010; Raver et al., 2013). Indeed, an increasing number of studies have provided evidence that both executive functions (EFs) (Blair and Razza, 2007; Nesbitt et al., 2013; Crook and Evans, 2014) and social competence (Murray and Malmgren, 2005; Bierman et al., 2008; Elias and Haynes, 2008) serve as pathways by which poverty-related risks can influence school readiness and academic achievement. EFs are higher order self-regulatory mechanisms that support planning and goal-directed behaviors that are important for daily life. Such processes include inhibitory control, cognitive flexibility, and working memory (Diamond, 2013). Social competence refers to prosocial skills that mediate positive interactions with others, such as peers and teachers (Raver and Zigler, 1997). While the prior literature has visibly demonstrated that both EFs and social competence are central to school readiness, prior studies have not examined the functional processes by which EFs and social competence *together* relate to poverty-related risk exposure and early academic performance.

Although considered distinct domains of development, mounting evidence suggests that EF and social competence skill acquisition might be functionally linked (Riggs et al., 2006). Indeed, a growing body of literature has explored how EF skills may influence the subsequent development of social competencies. Multiple studies have presented evidence that EF skills in the early academic grades predict social competence one to two years later (Riggs et al., 2004; Ciairano et al., 2007). In another noteworthy study, social competence mediated the relationship between EF and change in academic scores across one school year, as assessed in a sample of 7–12 years olds (Valiente et al., 2008). This same research team also found that social functioning, measured by social competence and behavioral functioning scores, mediated the relationship between effortful control at 6 years and academic scores at 12 years (Valiente et al., 2011). Additionally, peer acceptance has been shown to mediate the association between inhibitory control and math grades in a sample of 4th and 5th graders (Oberle and Reichl, 2013). Social-behavioral adjustment was also found to mediate the effect of EF on school readiness in a sample of preschoolers (Baptista et al., 2016). Similarly, another study utilizing a low-income sample demonstrated that inhibitory control predicted measures of social and emotional competence (Rhoades et al., 2009). Finally, the predictive effect of EF on social competence has been shown to span into adolescence, as evidenced by studies where EF prior to school entrance and/or during elementary school predicted social and academic competence in 6th graders (Jacobson et al., 2011) and 15–year olds (Holmes et al., 2016). It is possible that EFs are needed for engaging in prosocial interactions (e.g., maintaining focus, listening, inhibiting distractions or inappropriate behaviors, and mentalizing another's beliefs or emotions), and perhaps necessary for the development and maintenance of social competence skills and positive peer relationships (Bateson, 2005; Hughes and Ensor, 2007; Brock et al., 2009; Russ, 2016). Thus, it is possible that poverty-related disruption of EF development itself can underlie disparities in school readiness, but can further contribute to disparities in school readiness through disruption of social competence skill development.

Additional research has explored the concept that reciprocal paths, in which earlier social skills influence the subsequent development of EFs, may exist (Carlson, 2009; Lewis and Carpendale, 2009; Moriguchi, 2014; van Lier and Deater-Deckard, 2016). Indeed, it has long been theorized that humans develop higher mental functions within the context of interpersonal activity and social relationships (Luria, 1966; Vygotsky, 1978). For example, it has been proposed that various components of social interactions, such as communication with others, perspective-taking, and compliance to social norms, set the stage for developing and maintaining EFs (Vygotsky, 1978). This idea that EF development occurs via social interactions has been scientifically explored, primarily through the study of adult-child social interactions in scaffolding EFs (Landry et al., 2002; Bibok et al., 2009; Hughes and Ensor, 2009; Lewis and Carpendale, 2009; Blair et al., 2011; Roskam et al., 2014; de Wilde et al., 2016). However, a small but growing body of literature has assessed this in terms of peer interactions (Moriguchi, 2014; van Lier and Deater-Deckard, 2016). This body of literature has provided evidence that social interactions with peers are essential to developing self-regulation (Lindsey and Colwell, 2003) and EFs such as cognitive flexibility (Bateson, 2005) and inhibitory control (Peterson and Flanders, 2005). Moreover, experiencing problems with peers, as measured by items measuring peer rejection, victimization, and social exclusion has been shown to contribute to reduced EF skills later in childhood (Baumeister et al., 2002, 2005; Holmes et al., 2016). However, there is a lack of research regarding the predictive role of social competence, as assessed via prosocial skills and positive peer relationships, on the subsequent development of EFs, especially among at-risk populations. It is possible that social competence skills provide one with the ability to form and maintain positive, stable relationships, which in turn facilitate EF development by providing increased opportunities for social communication, perspective-taking, and adherence to social norms (Vygotsky, 1978; Baumeister et al., 2002, 2005). Thus, it is possible that the disruption of social competence skills can lead to a disruption of EF skill development and subsequent academic performance.

The present study aimed to identify developmental processes by which poverty-related risks can contribute to gaps in academic readiness and school achievement, by assessing the interplay between social competence and EFs across the first years of schooling. All data came from the Family Life Project, a prospective longitudinal research study following 1,292 children and families from birth, who are primarily of low socioeconomic status (Vernon-Feagans et al., 2013). Based on prior empirical and theoretical work, we hypothesized that EF and social competence would reciprocally influence each other across the early elementary school grades. More specifically, we hypothesized that EF through social competence, and social competence through EF would demonstrate cross-lagged mediation of the relationship between early-life poverty-related risk exposure and academic abilities in Grade 2 (G2). Thus, this study was designed to explore the developmental interrelations between EF and social competence as a function of poverty status, as it pertains to influencing early academic achievement. The present study bridges existing research regarding relations between early-life experience and EF/social competence with research on the significance of EF and social competence for school readiness. To the best of our knowledge, this is the first study to test reciprocal longitudinal paths between social competence and EF as mediators between poverty-related risk exposure and early academic abilities. Thus, it contributes to an expanding body of literature exploring the reciprocal development of EFs and prosocial behaviors as children transition to formal learning settings. In doing so, it expands the developmental scope and generalizability of the existing literature, by contributing longitudinal data regarding mediational processes involving EF and social competence in a predominantly socioeconomically disadvantaged sample.

## Materials and methods

### Participants

This study's data come from the Family Life Project, a prospective longitudinal study of children who were born from Fall 2003 to 2004. Participants were recruited from families residing in three low-income counties in Eastern North Caroline (NC) and three low-income counties in Central Pennsylvania (PA). These regions were carefully chosen to be representative of the Black South (NC) and Appalachia (PA). Low-income families were oversampled in both NC and PA, and African American families were oversampled in NC. Complex sampling procedures were used for recruitment, and the authors refer the reader to previously published materials for full details regarding the study design and sampling plan (Vernon-Feagans et al., 2013). The complete sample of the Family Life Project consisted of 1,292 families.

### Procedures

Demographic data come from a dataset collected in families' homes when the target children were approximately 2, 6, 15, 24, 36, and 58 months of age. Home visits were conducted by two trained research assistants assessing household characteristics, family demographics, child behavior, and parenting style in a mother-infant interaction task. EF data come from direct assessments conducted during the 36, 58-months and G1 home visits. Social competence data were collected at the Kindergarten (K) and Grade 1 (G1) school visits, via teacher completed questionnaires. Research assistants also visited children at their school in G2 and administered early academic achievement measures, including the Woodcock-Johnson III Tests of Achievement. Timepoints, measures, and age statistics for child EF, social competence, and academic outcome assessments are summarized in Table 1.

**Table 1:** Timepoints, measures, and ages of child executive function (EF), social competence, and academic outcome assessments.

**Timepoint**	**Measure**	***N***	**Mean age in months (*SD*)**
36 months	EF battery (Operation Span, Silly Sounds, Animal Go/No Go, Spatial Conflict, Something's the Same)	973	37.02 (0.14)
58 months	EF: Operation Span	983	60.28 (0.25)
	EF: Silly Sounds	995	60.37 (0.25)
	EF: Animal Go/No Go	980	60.39 (0.25)
	EF: Pick the Picture	1004	60.37 (0.25)
Kindergarten	Prosocial Behavior Subscale (Social Competence Scale)	985	71.63 (0.30)
	Prosocial Behavior Subscale (Strengths and Difficulties Questionnaire)	982	71.63 (0.30)
	Peer Relationships Scale	977	71.61 (0.30)
Grade 1	EF: Hearts and Flowers	991	83.57 (0.31)
	EF: Dimensional Change Card Sort	827	83.57 (0.31)
	EF: Backward Word Span	1033	83.52 (0.31)
	Prosocial Behavior Subscale (Social Competence Scale)	931	83.33 (0.30)
	Prosocial Behavior Subscale (Strengths and Difficulties Questionnaire)	929	83.33 (0.30)
	Peer Relationships Scale	929	83.33 (0.30)
Grade 2	Woodcock-Johnson III: Brief Reading	1044	95.35 (0.31)
	Woodcock-Johnson III: Applied Problems	1046	95.35 (0.31)

This study was reviewed and approved by the Institutional Review Board at Pennsylvania State University and the Office of Human Research Ethics at the University of North Carolina. Written informed consent was obtained from all adult participants and from the parents/legal guardians of all non-adult participants, in accordance with the Declaration of Helsinki. All participation was voluntary, with participants being informed prior to the study that they could remove their consent at any time.

### Measures

#### Covariates

State of residence (PA = 0, NC = 1) was used as a covariate in all analyses to control for site differences in variables. Additional demographic covariates were included in all analyses. Specifically, covariates were primary caregiver's report of child sex (Male = 0, Female = 1) and child race (not African American = 0; African American = 1) during the 2–months home visit, and age of the infant at the K data collection point.

All analyses also included levels of sensitive parenting as a covariate, to control for an indicator of positive social interactions with the primary caregiver during a free-play task. Parenting style was assessed during interactions between the primary caregiver and their infant during a 10 min semi-structured, free-play task during the 6-, 15-, 24-, and 36–months home visits. Caregivers were given instructions to play with their infant using a standardized set of toys. Highly trained coders scored mother-infant interactions from video recordings. Videos were coded for levels of caregivers' sensitivity, intrusiveness, detachment, stimulation, positive regard, and negative regard (National Institute of Child Health and Human Development Early Child Care Research Network, 1999; Cox and Crnic, Unpublished manuscript). Each dimension of behavior was coded from 1 (“not at all characteristic”) to 5 (“highly characteristic”). Principle factor analyses of parenting measures was conducted using an oblique rotation (i.e., Promax) for each time point. Dimensions included sensitive parenting (the average of stimulation, sensitivity, positive regard, animation, and detachment (reverse scored)) and negative parenting (average of instrusiveness, detachment, and negative regard), although only sensitive parenting was used for the current study (Mills-Koonce et al., 2011; Vernon-Feagans et al., 2013).

Lastly, as a robustness check, early-life EF was included as a covariate for all analyses. This EF data came from a commonly used battery of EF tasks, which was administered at the 36–months home visits. This battery was comprised of inhibitory control tasks, a working memory task, and an attention shifting task. The inhibitory control tasks included a Spatial Conflict task, an Animal Go/No-Go task, and a Silly Sounds task similar to the Stroop task. The working memory task was comprised of a Span-like task. The attention shifting task was essentially an item selection task, which was modeled after the Dimensional Change Card Sort task (Sulik et al., 2015). Using item response theory, which relies on a latent variable approach, an expected a posteriori (EAP) score was generated for each task. Full details about the computation of EAP scores and information regarding longitudinal measurement invariance of these scores has been previously described in detail elsewhere (Willoughby et al., 2012a). An average of EAP scores was computed to create a composite measure of EF ability at 36–months.

#### Cumulative risk

Poverty-related risk exposure was represented by computing a cumulative risk composite at 6, 15, 24, and 36 months from seven variables. These variables included family income-to-needs ratio, household density, neighborhood safety, maternal education, consistent partnership of a spouse/partner living in the home, maximum work hours of primary or secondary caregiver per week, and job prestige. Job prestige was coded using the National Opinion Research Center (NORC) coding system (Nakao and Treas, 1994). To create the continuous cumulative risk index, positively framed indicators were reverse-scored and each risk measure was standardized and averaged together, such that higher scores indicated higher risk (α = 0.82). As previously reported, this cumulative risk index was originally created through the assessment of nine social risk factors that were demographic indicators of socioeconomic status or had been related to poverty in prior research (Vernon-Feagans et al., 2013). These nine factors included family income-to-needs ratio, household density, neighborhood safety, maternal education, consistently partnered parents, employment hours, and job prestige, as well as maternal health, and ratings of food insufficiency. Principle component analyses at each age revealed a single dominant factor accounting for 33–36% of total variance among the nine indicators (Vernon-Feagans et al., 2013). With the exception of maternal health and ratings of food insufficiency, all indicators loaded on this factor. As an additional strategy, a within-family mean score was also computed from the repeated measures of each of the nine risk variables from the 6, 15, 24, and 36 months visits. These across-time mean risk scores were moderately correlated with one another, with the exception of maternal health and ratings of food insufficiency. Furthermore, a principle components analysis of these across-time mean risk scores revealed a single dominant factor accounting for 39% of total variance among the nine indicators. Again, the same seven indicators loaded substantially on this main risk index (income-to-needs ratio, household density, neighborhood safety, maternal education, consistenly partnered parents, employment hours, and job prestige) (Vernon-Feagans et al., 2013). Both strategies of analyses support that early poverty-related risk can be represented by a single summary variable computed using repeated measures of risk indicators. For the present analyses, it was chosen to use the cumulative risk index as calculated from the across-time mean risk indicators because it was the most parsimonious measure.

#### Social competence

Children's social competence was modeled as a latent factor comprised of teachers' ratings across three scales from three measures. Specifically, we used the Prosocial subscale from the *Strengths and Difficulties Questionnaire* (SDQ) (Goodman, 1997), the Prosocial subscale from the *Social Competence Scale* (Dodge et al., 1994), and the *Peer Relationships Scale* (Ladd and Profilet, 1996) as observed indicators.

During the K and G1 school visits, teachers completed the SDQ, a 25–item questionnaire for use with children aged 3-16 years old. Items were rated on a 3 point scale as not true (zero), somewhat true (one point), or certainly true (two points). The SDQ has five subscales (Emotional Symptoms, Conduct Problems, Hyperactivity/Inattention, Prosocial Behavior, and Peer Relationship Problems), however the current study used only the Prosocial Behavior subscale. The Prosocial subscale measures the amount of prosocial characteristics displayed by a child (e.g., “shares readily with other children”), with higher scores representing higher levels of prosocial behaviors. A comprehensive review has been previously published, indicating that the teacher-completed SDQ has satisfactory internal consistency (α = 0.70), as well as test-retest reliability (*r* = 0.77) for these scales (Stone et al., 2010). The Prosocial subscale from the *Social Competence Scale* was also completed by teachers. This subscale comprises four Likert-type items where children are rated in terms of their ability to successfully interact with others (e.g., “resolves problems with other children on his or her own,” “cooperates”). Items were rated on a scale from “almost never” (one point) to “almost always” (six points) with higher ratings indicating higher social competence. This scale shows acceptable internal consistency (α = 0.93). Finally, teachers completed the *Peer Relationships Scale*, comprised of six Likert-type items rated from “almost never” (zero points) to “almost always” (five points) (e.g., “is liked by classmates”). Negatively framed items (e.g., “is teased or picked on by classmates”) were reverse-scored to create a positively framed indicator of peer relationships.

#### Executive function (EF)

EF was assessed at the 58 months and G1 home visits (Table 2). At 58 months, EF was measured using a common battery of EF tasks. The EF battery included inhibitory control, working memory, and attention shifting tasks. The inhibitory control tasks were a Silly Sounds task modeled after the Stroop task, and an Animal Go/No-Go task (Durston et al., 2002). The working memory task was an Operation Span task based on the work of Engle (2002) (Engle, 2002). The attention shifting task was a Pick the Picture item selection task modeled after the Flexible Item Selection Task (Jacques and Zelazo, 2001; Willoughby et al., 2011). These tasks are described in Table 2 and are further described and evaluated in detail elsewhere (Willoughby et al., 2010, 2012b; Willoughby and Blair, 2011). EAP scores were generated for every task and equated across time using a calibration sample. Using EAP scores for each task, EF was modeled as a latent variable at 58 months.

**Table 2:** Executive function (EF) tasks at 58 months and Grade 1.

**Timepoint**	**Measure**	**Description**
58 months	Operation Span	Children were shown an outline of a house containing a drawing of an animal and a colored dot. Children were asked to name the animal and the color. Children were then shown the outline of the house again on a subsequent page and asked to recall which animal was in the house.
	Silly Sounds	In this Stroop-like task, children were instructed to make the sound of a cat when presented with an image of a dog, and the sound of a dog when presented with an image of a cat.
	Animal Go/No Go	This is a standard go/no go task presented in a flipbook format. Children were instructed to click a large button every time they were presented with an image of an animal, except when an image of a pig was presented.
	Pick the Picture	In this self-ordered pointing task, children were presented with a set of pictures and instructed to pick each picture so that each of the pictures “gets a turn.” For example, for a set of pictures containing an apple and a cat, if the child picks the apple, they must then pick the cat on the next page. The arrangement of pictures within each set was randomized so that children could not rely on spatial information.
Grade 1	Hearts and Flowers	Children were instructed to push buttons on a keyboard based on the image (heart or flower), and location of image on the keyboard's screen. For hearts, children were told to push a button on the same side of the keyboard as the heart's location on the screen. For flowers, children were told to push a button on the opposite side of the keyboard as the flower's location on the screen.
	Dimensional Change Card Sort	Children were asked to shift between sorting a set of cards by their shape or color, first by one feature (e.g., shape), and then by the other feature (e.g., color). Lastly, children were instructed to sort cards according to an audio cue played at the beginning of each trial identifying the feature by which they should sort.
	Backward Word Span	Children instructed to repeat a list of familiar, single-syllable and related words in reverse order (e.g., repeat “book, cup” as “cup, book”).

At G1, EF was modeled as a latent variable comprised of the Hearts and Flowers task, a measure of inhibition and attention shifting (Davidson et al., 2006), a Backward Word Span task assessing working memory (Davis and Pratt, 1996), and a Dimensional Change Card Sort task (Willoughby et al., 2011). For the Hearts and Flowers task, a target picture (heart or flower) was presented on one side of a laptop screen. Children were told to push a button on the keyboard's laptop that corresponded with the picture's location on the display. For hearts, children were told to push the button on the same side of the target picture. For flowers, children were told to push the button on the opposite side of the target picture. Children were allowed instructional and practice trials, which could be repeated up to three times, if needed. Instructional/practice trials were followed by 20 hearts-only trials, 12 flowers-only trials, and 33 mixed trials that included hearts and flowers. Children's accuracy was measured, with accuracy for the intermixed section of the task being used for the current study's purposes.

For the Backward Word Span task children were requested to repeat a list of familiar single-syllable and related words (but not semantically related) in reverse order (i.e., if the instructor says “book, cup,” the child is to say “cup, book”). Children were allowed instructional and practice trials, which could be repeated up to three times, if needed. For the task, the list of words to be repeated increased with every successful trial over the course of six trials. The task concluded when the child made three consecutive errors, with their score being recorded as the highest number of words correctly recalled.

For the Dimensional Change Card Sort task, children were asked to sort cards by two features, shape or color. In the first segment of the task, children were told to sort cards by either shape or color. In the second segment, children were told to sort cards by the other feature. In the third and final segments, trials were mixed such that children were told to sort 50 cards according to an audio cue played at the beginning of each trial identifying the feature by which they should sort. The percentage of correct responses during these mixed trials was used for the current study's purposes.

#### Academic outcomes

Academic Outcomes was modeled as a latent variable of skills related to reading and math, which were evaluated during the G2 school visits via the Woodcock-Johnson III Tests of Achievement. The Woodcock-Johnson III Tests of Achievement is a norm-referenced battery of subtests used to measure scholastic aptitude, spoken language, and academic achievement (Woodcock et al., 2001). The Brief Reading subtest was used as a measure of reading achievement, while the Applied Problems subtest was used as a measure of math achievement. The validity and reliability of the Woodcock-Johnson III Tests of Achievement have been previously established and elaborated on elsewhere (Woodcock et al., 2001).

### Missing data

The entire study sample consisted of 1,292 families at the 2–months home visit, with 1,204 children seen at 6–months postpartum, 1,169 children at 15–months, 1,144 children at 24–months, 1,123 children at 36–months, 1,099 at 58 months, 1,063 at K, 1,093 at G1, and 1,049 at G2. Complete data were available for sex and race of the target child, as well as state of residence. Participants were included in the analysis if they were not missing data for one or more assessments of the Woodcock-Johnson III Tests of Achievement. This resulted in an analytic sample of *N* = 1,044, which was used for all analyses. To assess possible differential attrition, variables for which there was complete information were examined, including race, sex, child at age K, and cumulative risk, with few variables indicating differences between children who were present vs. missing at each data collection time point. Specifically, child race and age demonstrated differences, such that children not included in our analyses were more likely to be African American and older. Complete information related to missing data is available upon request to the first author. Full information maximum likelihood (FIML) estimation was used to reduce bias in estimates related to missing data (Enders, 2010).

### Statistical analysis

All data were tabulated and statistically analyzed using IBM SPSS Statistics Version 21.0, and Mplus softwares. Descriptive analyses and bivariate correlations were computed for all study variables in SPSS 21.0. Structural equation models testing autoregressive cross-lagged mediation were estimated using the bootstrapping procedure (5,000 bootstraps) (Shrout and Bolger, 2002) in Mplus 7 software (Muthén and Muthén, 1998-2012). All parameter estimates are reported as standardized effects (Preacher and Hayes, 2008). Model fit was evaluated using the Comparative Fit Index (CFI), the root mean squared error of approximation (RMSEA), and the standardized root square mean residual (SRMR) fit indices, with CFI ≥ 0.95, RMSEA < 0.05, and SRMR < 0.08 being indicative of good fit (Kline, 2015). The raw data of this manuscript will be made available by the authors to any qualified researcher, without reservation.

## Results

### Preliminary analysis

**Figure 1:**
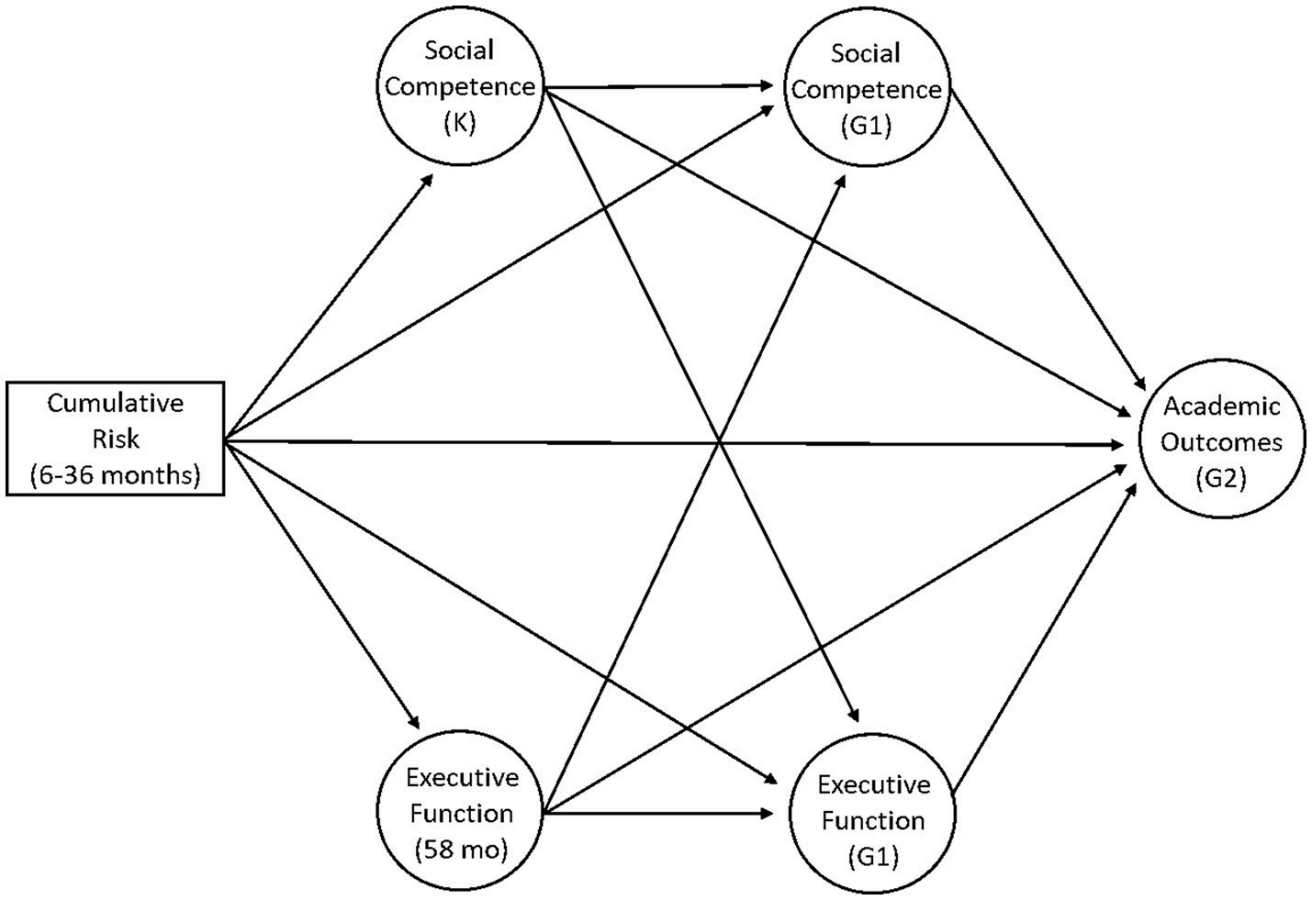
Hypothesized autoregressive cross-lagged mediation model relating poverty-related cumulative risk (6–36 months), social competence (K & G1), executive function (58 months & G1), and academic outcomes (G2). K, Kindergarten; G1, Grade 1; G2, Grade 2; mo, months. Covariates are not shown.

Descriptive statistics and bivariate correlations among all study variables appear in Tables 3 and 4, respectively. The overall pattern of correlations was consistent with the study's hypothesis. Specifically, our independent variable (poverty-related cumulative risk exposure) was negatively associated with our proposed mediating variables: Cumulative Risk was negatively associated with each observed indicator of social competence at K and G1, as well as EF at 58 months and G1. Furthermore, our proposed mediating variables were positively associated with academic outcomes at G2: Social behavior measures at K and G1 were positively related to Brief Reading and Applied Problems at G2. EF measures were also positively related to Brief Reading and Applied Problems at G2. Additionally, Cumulative Risk was negatively associated with Brief Reading and Applied Problems at G2. Finally, our proposed mediating variables were positively associated with each other: Social behavior measures at K and G1 were positively related to EF at 58 months and EF at G1. Together, this pattern of significant associations provided support for our hypothesized mediating model (Figure 1), which was tested below.

**Table 3:** Descriptive statistics for observed variables.

	***N***	**Mean or %**	***SD***	**Minimum**	**Maximum**
Race (% African American)	1292	42%	–	–	–
Sex (% Male)	1292	51%	–	–	–
State of Residence (% NC)	1292	60%	–	–	–
Child Age at K (years)	1072	5.99	0.31	5.40	7.35
Cumulative Risk (6–36 months)	1235	−0.01	0.69	−2.66	2.19
Sensitive Parenting (6–36 months)	1221	2.85	0.66	1.00	4.65
Prosocial Behavior SCS (K)	985	4.33	1.15	1.00	6.00
Prosocial Behavior SDQ (K)	982	1.54	0.47	0.00	2.00
Peer Relationships (K)	977	3.40	0.61	0.50	4.00
Prosocial Behavior SCS (G1)	931	4.27	1.18	1.00	6.00
Prosocial Behavior SDQ (G1)	929	1.52	0.49	0.00	2.00
Peer Relationships (G1)	929	3.34	0.66	0.00	4.00
EF (36 months)	973	−0.54	0.54	−1.98	1.18
Operation Span (58 months)	983	0.34	0.68	−1.71	1.98
Silly Sounds (58 months)	995	0.21	0.78	−1.98	1.41
Animal Go/No Go (58 months)	980	0.28	0.69	−1.98	0.85
Pick the Picture (58 months)	1004	0.28	0.82	−2.54	2.25
H&F % correct (G1)	991	0.80	0.18	0.15	1.00
DCCS % correct (G1)	827	0.85	0.14	0.25	1.00
Backward Word Span (G1)	1033	2.43	0.74	1.00	5.00
WJ: Brief Reading (G2)	1044	471.60	22.53	347.00	518.00
WJ: Applied Problems (G2)	1046	472.43	20.85	318.00	536.00

*K, Kindergarten; G1, Grade 1; G2, Grade 2; SCS, Social Competence Scale; SDQ, Strengths & Difficulties Questionnaire; EF, Executive Function; H&F, Hearts & Flowers; DCCS, Dimensional Change Card Sort; WJ, Woodcock Johnson*.

**Table 4:** Correlations among observed variables.

	**1**	**2**	**3**	**4**	**5**	**6**	**7**	**8**	**9**	**10**	**11**	**12**	**13**	**14**	**15**	**16**	**17**	**18**	**19**	**20**	**21**
1. Race	1																				
2. Sex	−0.01	1																			
3. State of Residence	0.60^**^	−0.08^**^	1																		
4. Child Age at K	−0.19^**^	0.02	−0.07^*^	1																	
5. Cumulative Risk (6–36 months)	0.44^**^	−0.03	0.26^*^	−0.07^*^	1																
6. Sensitive Parenting (6–36 months)	−0.41^**^	−0.02	−0.29^*^	0.07^*^	−0.59^**^	1															
7. Prosocial Behavior SCS (K)	−0.10^**^	−0.10^**^	0.01	0.12^**^	−0.19^**^	0.18^**^	1														
8. Prosocial Behavior SDQ (K)	−0.10^**^	−0.12^**^	0.03	0.11^**^	−0.20^**^	0.19^**^	0.72^**^	1													
9. Peer Relationships (K)	−0.06^**^	0.00	0.03	0.12^**^	−0.20^**^	0.17^**^	0.67^**^	0.63^**^	1												
10. Prosocial Behavior SCS (G1)	−0.19^**^	−0.19^**^	−0.06	0.09^**^	−0.22^**^	0.23^**^	0.46^**^	0.43^**^	0.37^**^	1											
11. Prosocial Behavior SDQ (G1)	−0.18^**^	−0.19^**^	−0.05	0.07^*^	−0.23^**^	0.22^**^	0.37^**^	0.42^**^	0.34^**^	0.76^**^	1										
12. Peer Relationships (G1)	−0.17^**^	−0.09^**^	−0.05	0.06	−0.21^**^	0.23^**^	0.39^**^	0.40^**^	0.41^**^	0.67^**^	0.68^**^	1									
13. EF (36 months)	−0.30^**^	−0.09^**^	−0.30^*^	0.00	−0.29^**^	0.30^**^	0.12^**^	0.09^**^	0.10^**^	0.17^**^	0.15^**^	0.16^**^	1								
14. Operation Span (58 months)	−0.09^**^	−0.03	−0.08^*^	0.07^*^	−0.13^**^	0.15^**^	0.11^**^	0.06	0.09^**^	0.12^**^	0.12^**^	0.13^**^	0.13^**^	1							
15. Silly Sounds (58 months)	−0.13^**^	−0.15^**^	−0.08^*^	0.03	−0.20^**^	0.17^**^	0.17^**^	0.17^**^	0.17^**^	0.17^**^	0.18^**^	0.18^**^	0.13^**^	0.17^**^	1						
16. Animal Go/No Go (58 months)	−0.07^*^	−0.19^*^	−0.06	0.01	−0.10^**^	0.15^**^	0.22^**^	0.20^**^	0.19^**^	0.22^**^	0.24^**^	0.24^**^	0.18^**^	0.15^**^	0.21^**^	1					
17. Pick the Picture (58 months)	−0.14^**^	−0.12^**^	−0.07^*^	0.08^**^	−0.24^**^	0.26^**^	0.19^**^	0.17^**^	0.16^**^	0.24^**^	0.21^**^	0.26^**^	0.26^**^	0.24^**^	0.28^**^	0.27^**^	1				
18. H&F % correct (G1)	−0.22^**^	−0.04	−0.18^*^	0.13^**^	−0.26^**^	0.29^**^	0.22^**^	0.14^**^	0.19^**^	0.24^**^	0.20^**^	0.23^**^	0.24^**^	0.19^**^	0.18^**^	0.21^**^	0.25^**^	1			
19. DCCS % correct (G1)	−0.09^*^	−0.11^**^	0.02	0.10^**^	−0.12^**^	0.14^**^	0.17^**^	0.13^**^	0.13^**^	0.16^**^	0.11^**^	0.11^**^	0.13^**^	0.12^**^	0.17^**^	0.16^**^	0.17^**^	0.38^**^	1		
20. Backward Word Span (G1)	−0.18^**^	−0.05	−0.09^*^	0.10^**^	−0.23^**^	0.23^**^	0.16^**^	0.14^**^	0.11^**^	0.18^**^	0.15^**^	0.19^**^	0.16^**^	0.13^**^	0.17^**^	0.13^**^	0.18^**^	0.25^**^	0.20^**^	1	
21. WJ: Applied Problems (G2)	−0.31^**^	0.04	−0.20^*^	0.14^**^	−0.37^**^	0.38^**^	0.28^**^	0.24^**^	0.22^**^	0.33^**^	0.29^**^	0.29^**^	0.29^**^	0.23^**^	0.26^**^	0.21^**^	0.34^**^	0.43^**^	0.33^**^	0.41^**^	1
22. WJ: Brief Reading (G2)	−0.18^**^	−0.10^**^	−0.10^*^	0.09^**^	−0.37^**^	0.31^**^	0.31^**^	0.26^**^	0.22^**^	0.33^**^	0.28^**^	0.28^**^	0.23^**^	0.18^**^	0.25^**^	0.21^**^	0.31^**^	0.38^**^	0.26^**^	0.42^**^	0.69^**^

** *p < 0.01*,

* *p < 0.05; K, Kindergarten; G1, Grade 1; G2, Grade 2; SCS, Social Competence Scale; SDQ, Strengths & Difficulties Questionnaire; EF, Executive Function; H&F, Hearts & Flowers; DCCS, Dimensional Change Card Sort; WJ, Woodcock Johnson*.

### Structural equation modeling

To examine relationships between Cumulative Risk, measures of social competence and EF, we used structural equation modeling. We modeled Social Competence as a latent variable at K and G1 with the Prosocial subscales of the SCS and SDQ, as well as the Peer Relationships Scale as indicators. Similarly, we modeled EF at 58 months and G1 as a latent variable with inhibitory control, working memory, and attention set shifting tasks as indicators. Lastly, we modeled Academic Outcomes at G2 using a latent variable with the Brief Reading and Applied Problems as indicators. We included observed variables for state of residence, race of child, sex of child, age of child, sensitive parenting (6–36 months), and EF at 36 months as covariates. Additionally, observed variables of family income-to-needs ratio, household density, neighborhood safety, maternal education, consistent partnership of a spouse or partner, maximum work hours of primary or secondary caregiver per week, and job prestige were used to compute a poverty-related Cumulative Risk index.

#### Measurement model

Prior to addressing our primary research question, we evaluated our measurement model that included the following latent variables: Social Competence at K, Social Competence at G1, EF at 58 months and G1, and Academic Outcomes at G2. This measurement model fit the data well: CFI = 0.99; RMSEA = 0.030 (90% confidence interval [0.023, 0.037]); SRMR = 0.027. Factor loadings of observed indicators on latent variables are reported in Table 5. Parameter estimates demonstrated that loadings were statistically significant in the expected direction, and that all latent variances were statistically significant. The correlation between EF and Social Competence latent variables was relatively high for both the K/58 months time point (ϕ = 0.42) and G1 time point (ϕ = 0.43).

**Table 5:** Loadings of observed indicators on latent variables.

**Latent variable**	**Indicators**	**β**
Social Competence (K)	Prosocial subscale (SDQ)	0.82
	Prosocial subscale (SCS)	0.87
	Peer Relationships Scale	0.77
Social Competence (G1)	Prosocial subscale (SDQ)	0.86
	Prosocial subscale (SCS)	0.87
	Peer Relationships Scale	0.80
Executive Function (58 months)	Operation Span	0.40
	Silly Sounds	0.49
	Animal Go/No Go	0.51
	Pick the Picture	0.62
Executive Function (G1)	H&F	0.61
	Backward Word Span	0.53
	DCCS	0.51
Academic Abilities (G2)	WJ: Brief Reading	0.80
	WJ: Applied Problems	0.86

*All coefficients are standardized and significant at p < 0.0001. K, Kindergarten; G1, Grade 1; G2, Grade 2; SCS, Social Competence Scale; SDQ, Strengths and Difficulties Questionnaire; H&F, Hearts & Flowers; DCCS, Dimensional Change Card Sort; WJ, Woodcock Johnson*.

Before testing mediation, we also evaluated independent, direct associations with early-life Cumulative Risk predicting Social Competence in K and G1, EF at 58 months and G1, and academic outcomes in G2. As expected, Cumulative Risk negatively predicted Academic Outcomes (β = −0.220, *SE* = 0.046, *p* < 0.001). Cumulative Risk also negatively predicted EF at 58 months (β = −0.169, *SE* = 0.049, *p* = 0.001) and G1 (β = −0.165, *SE* = 0.052, *p* = 0.002). Finally, Cumulative Risk negatively predicted Social Competence at K (β = −0.162, *SE* = 0.044, *p* < 0.001) and G1 (β = −0.123, *SE* = 0.045, *p* = 0.006).

#### Structural model

Figure 1 displays our hypothesized structural model (without covariates). In this model, we estimated an autoregressive cross-lagged mediation model that included all variables of interest. Specifically, we examined direct effects of all variables of interest on Academic Outcomes (G2), EF (58 months and G1), and Social Competence (K and G1). Furthermore, we examined indirect effects of Cumulative Risk on Academic Outcomes through EF (58 months, G1), or Social Competence (K, G1) separately to determine the extent to which these variables mediate effects of poverty-related Cumulative Risk on child early Academic Outcomes. Indirect effects of Cumulative Risk on Academic Outcomes through EF at 58 months through EF at G1, as well as through Social Competence at K through Social Competence at G2 were also examined. Finally, indirect effects of Cumulative Risk on Academic Outcomes through EF (58 months) through Social Competence (G1) (and vice versa: through Social Competence [K] through EF [G1]) were examined. As previously, we controlled for our covariates on each endogenous variable in the model.

**Figure 2:**
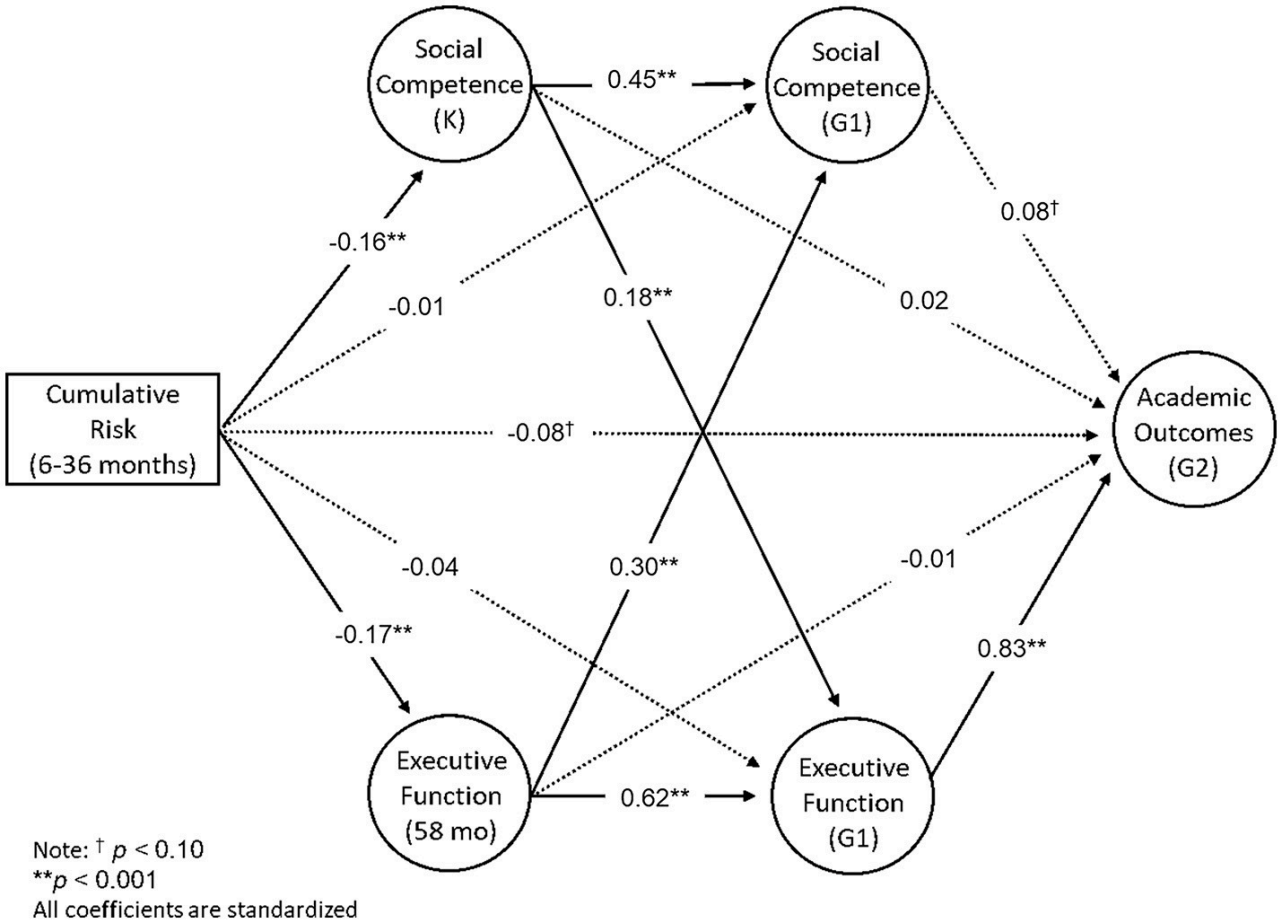
Autoregressive cross-lagged mediation model relating poverty-related cumulative risk (6–36 months), social competence (K & G1), executive function (58 months & G1), and academic outcomes (G2). All coefficients are standardized (β). All significant pathways are bolded. **p < 0.001, ^†^*p* < 0.10, K, Kindergarten; G1, Grade 1; G2, Grade 2; mo, months. Covariates are not shown.

The observed structural model fit the data well: CFI = 0.96; RMSEA = 0.039 (90% confidence interval [0.035, 0.044]); SRMR = 0.041 (Figure 2). All coefficients presented are standardized estimates and thus reflect changes in standard deviations. Direct effects are reported in Table 6. Of particular note, Cumulative Risk was not directly associated with Social Competence or EF at G1, but was negatively associated with Social Competence at K and EF at 58 months. Furthermore, EF at G1 was negatively associated with Academic Outcomes. However, Social Competence at K and G1, as well as EF at 58 months were not significantly associated with Academic Outcomes at G2. Social Competence at K was positively associated with EF and Social Competence at G1. EF at 58 months was also positively associated with EF and Social Competence at G1.

**Table 6:** Direct effects of models predicting social competence, executive function, and academic achievement.

	**Social competence (K)**			**Social competence (G1)**			**EF (58 months)**			**EF (G1)**			**Academic outcomes (G2)**		
**Predictor**	**β**	***SE***	***p***	**β**	***SE***	***p***	**β**	***SE***	***p***	**β**	***SE***	***p***	**β**	***SE***	***p***
Cumulative Risk (6–36 months)	−0.16^**^	0.05	0.00	−0.01	0.04	0.96	−0.17^**^	0.05	0.00	−0.04	0.06	0.48	−0.08^†^	0.05	0.09
Sex	−0.09^*^	0.04	0.01	−0.08^*^	0.03	0.02	−0.23^**^	0.04	0.00	0.08	0.05	0.10	0.10^*^	0.04	0.02
Child Age at K	0.13^**^	0.03	0.00	−0.02	0.03	0.49	0.06	0.04	0.14	0.09^†^	0.05	0.06	−0.02	0.04	0.56
Race	−0.08	0.05	0.10	−0.12^*^	0.05	0.01	0.02	0.06	0.71	−00.09	0.06	0.14	0.01	0.06	0.88
State of Residence	0.20^**^	0.04	0.00	0.02	0.04	0.62	0.04	0.05	0.45	0.04	0.05	0.44	−0.05	0.05	0.27
Sensitive Parenting (6–36 months)	0.10^*^	0.04	0.02	0.03	0.04	0.43	0.22^**^	0.05	0.00	0.07	0.06	0.28	0.00	0.05	0.10
EF (36 months)	0.10^*^	0.05	0.02	−0.04	0.04	0.29	0.27^**^	0.05	0.00	0.04	0.05	0.43	−0.01	0.05	0.83
EF (58 months)	–	–	–	0.30^**^	0.06	0.00	–	–	–	0.62^**^	0.08	0.00	−0.01	0.13	0.96
EF (G1)	–	–	–	–	–	–	–	–	–	–	–	–	0.83^**^	0.14	0.00
Social Competence (K)	–	–	–	0.45^**^	0.04	0.00	–	–	–	0.18^**^	0.05	0.00	0.02	0.06	0.79
Social Competence (G1)	–	–	–	–	–	–	–	–	–	–	–	–	0.08^†^	0.05	0.08
*R*^2^	0.12	0.02	0.00	0.40	0.04	0.00	0.31	0.04	0.00	0.58	0.07	0.00	0.80	0.07	0.00

* *p < 0.05*,

** *p < 0.01*,

† *p < 0.10; All coefficients are standardized. EF, Executive Function; K, Kindergarten; G1, Grade 1; G2 = Grade 2*.

### Cross-lagged mediation

Our primary hypothesis was that EF (58 months) through Social Competence (G1), and Social Competence (K) through EF (G1) would demonstrate cross-lagged mediation of the relationship between early-life cumulative poverty-related risk exposure and academic abilities in Grade 2 (G2). To evaluate this hypothesis, we tested the indirect effects of Cumulative Risk (6–36 months) on Academic Outcomes at G2. Specifically, we tested indirect effects via serial pathways involving Social Competence (K and G1) and EF (58 months & G1). We also tested indirect paths between Cumulative Risk and Academic Outcomes through Social Competence and EF separately.

Table 7 displays the mediation results and indicates that Social Competence at K mediated the effects of Cumulative Risk on Academic Outcomes through EF at G1, with this pathway mediating 16% of the total effect. Additionally, EF at 58 months via EF at G1 significantly mediated the relationship between Cumulative Risk on Academic Outcomes, with this pathway mediating 36% of the total effect. Thus, Social Competence at K through EF at G1 was a significant mediating pathway even when accounting for mediation via EF at K through EF at G1. Furthermore, these significant mediating pathways occurred while controlling for EF at 36 months, which was a covariate in our model. However, EF at 58 months through Social Competence at G1 did not demonstrate cross-lagged mediation of the effects of Cumulative Risk on Academic Outcomes. Additionally, mediational pathways involving EF or Social Competence as independent mediators were not significant.

**Table 7:** Indirect effects of mediating pathways predicting academic outcomes.

	**Academic Outcomes G2**		
**Predictor**	β	***SE***	**95% Confidence Interval**
Cumulative Risk → Social Competence (K)	−0.002	0.009	(−0.015, 0.014)
Cumulative Risk → Social Competence (G1)	0.001	0.004	(−0.007, 0.005)
Cumulative Risk → EF (58 months)	0.001	0.022	(−0.027, 0.047)
Cumulative Risk → EF (G1)	−0.032	0.049	(−0.114, 0.040)
Cumulative Risk → Social Competence (K) → Social Competence (G1)	−0.005	0.003	(−0.012, 0.000)
Cumulative Risk → EF (58 months) → Social Competence (G1)	−0.004	0.003	(−0.008, 0.000)
Cumulative risk → EF (58 months) → EF (G1)	−0.085^*^	0.035	(−0.151, −0.038)
Cumulative Risk → Social Competence (K) → EF (G1)	−0.024^*^	0.012	(−0.046, −0.009)

* *Significant (confidence interval does not contain zero). All coefficients are standardized. EF, Executive Function; K, Kindergarten; G1, Grade 1, G2, Grade 2*.

## Discussion

This study aimed to extend existing literature regarding potential developmental processes by which growing up in poverty can influence school readiness and academic achievement. Environmental influences on the development of EF and social competence, and the importance of EF and social competence for school readiness are emerging areas of research. However, no prior studies have sought to bridge these areas of research by exploring autoregressive cross-lagged mediational pathways involving social competence and EF development as it relates to cumulative poverty-related risk and academic outcomes.

Here we tested the primary hypothesis that the development of EF and social competence reciprocally influence each other, such that EF through social competence (and vice versa) demonstrate cross-lagged mediation of poverty-related risk exposure and early academic abilities. Our findings provided partial support for this hypothesis, by revealing that social competence at K through EF at G1 significantly mediates the effect of poverty-related cumulative risk exposure on G2 academic outcomes. More specifically, challenges in social competence at K associated with poverty-related risks negatively impacted academic abilities related to math and literacy skills *through* challenges in EF at G1. Importantly, our conservatively specified model allows for strong inference that social competence is meaningfully related to academic outcomes through EF at G1. Furthermore, our model demonstrated significant longitudinal mediation of the effect of cumulative risk on academic outcomes via EF at 58 months through EF at G1. However, our hypothesis was not fully supported, as the reciprocal pathway by which EF at K through social competence at G1 mediates the relationship between cumulative risk and academic outcomes was merely approaching significance.

These results converge with a growing literature indicating the importance of EF development for promoting early academic competence (Blair, 2002; Blair and Razza, 2007; Bierman et al., 2008; Willoughby et al., 2012b). Furthermore, these findings suggest that the development of EFs might be influenced by and/or functionally linked to the development of social competence, at least across early development. That is, our present findings suggest that disrupted social competence may influence the development of EF with implications for academic achievement in the early elementary grades. These findings are consistent with empirical research linking EF and social development (de Wilde et al., 2016; Holmes et al., 2016), as well as theoretical work regarding the interplay between EF and social development (Vygotsky, 1978; Riggs et al., 2006; Moriguchi, 2014). For example, both empirical and theoretical work converge on findings that positive, stable relationships are an active ingredient in facilitating cognitive development and maintaining higher-order cognitive abilities across the lifespan (Vygotsky, 1978; Baumeister et al., 2002, 2005).

Thus, our causal theory of relations between social competence and EF is based in prior empirical and theoretical work demonstrating that successful prosocial interactions require many tasks that rely on EFs, such as mentalizing another's ever-changing beliefs, expectations, and emotions, as well as maintaining focus, problem-solving and inhibitory control of inappropriate behaviors (Hughes and Ensor, 2007; Brock et al., 2009). In other words, successful prosocial interactions that are reliant upon social competence skills may serve as important, and perhaps even necessary, opportunities for continued practice and development of EF skills (Bateson, 2005; Russ, 2016). Such a conceptual model is consistent with long-standing approaches to the interrelation of social and cognitive development based on the theory of Vygotsky (Cole, 1995). However, in our conservatively specified model including early measures of EF and social competence, the mediational pathway of EF at 58 months through social competence at G1 was only approaching significance, and thus did not display strong convergence with prior work not including the earlier time points for these measures (Valiente et al., 2008, 2011). In our analysis, the effect of EF at 58 months on academic ability in G2 was mediated through EF measured in G1.

A goal of future research should be determining the mechanisms that underlie the developmental relations between social competence and EF development. From a developmental neuroscience perspective, previous studies have suggested that the development of the medial prefrontal cortex (mPFC) is functionally involved in emerging EF abilities, with structural variations mediating the relationship between early-life stress and EF skills (Arnsten and Li, 2005). While social skills are subserved by a diffuse, integrated neural network (McCabe et al., 2001; Decety et al., 2004), pathology of frontal regions has been associated with social difficulties (Rosema et al., 2012). Thus, disruption of social competence and EF, such as via socioeconomic disadvantage and its related stressors, may share altered mPFC development as a common underlying mechanism. Indeed, the mPFC has prolonged postnatal development, and is thus more susceptible to environmental influences (Noble et al., 2007; Kishiyama et al., 2009; Hackman et al., 2010).

The present study also provides novel, preliminary support for the premise that social competence through EF is a pathway by which poverty-related cumulative risk predicts early academic competence in a high-risk, low-income sample. The disruption of social development, such as via exposure to poverty-related stressors, may have a profound sequential effect on EF, serving as a causal pathway leading to broader achievement inequalities. These findings are consistent with, and extend, previous studies highlighting the importance of social competence and EF, individually, as important markers of school readiness (Campbell and Stauffenberg, 2008; Blair and Raver, 2015; Eickmann et al., 2016). Children with social skill problems participate less in classroom activities and social relations, have lower quality relationships with teachers and peers, and have decreased abilities in planning and completing academic tasks (Blair, 2002; de Wilde et al., 2016). Furthermore, it has been proposed that adverse social experiences and/or social stress as a result of poor social competence can impair regulatory resources, leading to a monopolization of EFs and impairment of cognitive functions (Baumeister et al., 2002; Davies et al., 2008). Consequently, problems with social competence and EF skills place children at risk of academic difficulties. However, the previous research has largely failed to consider the processes by which social and EF development, considered together, affect school readiness and academic performance, especially for at-risk children growing up in poverty. Here, by exploring possible mediational paths related to both EF and social development, we have provided evidence furthering the understanding of the longitudinal processes by which cumulative risk can influence academic ability.

The negative associations between poverty-related cumulative risk and social competence/EF development likely results from exposure to stressors that disrupt skill development. Growing up in conditions of low socioeconomic status affects children's outcomes in a multitude of ways. Low socioeconomic status families face significant economic and housing obstacles, among other barriers, that can diminish parent-infant relationship quality and socialization opportunities for children (Ryan et al., 2006). Disruptions in early social and emotional experiences, such as decreased sensitive and responsive caregiving, may set the stage for subsequent social competence and EF challenges. For example, low rates of parental sensitivity decrease opportunities for scaffolding of social behavior and emotional regulation, language exchange, and sustained joint attention (Goldsmith and Rogoff, 1997; Lengua et al., 2007).

Altogether, this study provided us with the opportunity to explore potential mechanisms regarding the impact of early-life risk exposure on academic ability. Additionally, the use of autoregressive cross-lagged mediation provided the opportunity to identify how social competence and EF skills influence each other across development, serving as indirect pathways by which poverty-related risks might influence early academic abilities. Such methodology provides an important first step for understanding complex processes by which poverty-related stressors impacts child development, which can in turn inform preventative and remediating intervention efforts.

A major strength of this study was the use of a longitudinal prospective design, which allowed for a clearer definition of the relationship between cumulative poverty-related risk and the development of academic abilities in a large sample size (*N* = 1,292). Such design provides stronger evidence for causal order than cross-sectional designs. However, the current findings should be interpreted with the following limitations in mind. While exploring autoregressive cross-lagged mediation serves as an important first step for understanding potential complex processes and mechanisms underlying the relationship between cumulative risk and academic abilities, our analysis is based on correlative data. Human studies are fundamentally limited in the extent that they can establish causal mechanisms by which poverty-related adversity influences development (Perry et al., 2018). However, it is important to note that our in-school and in-home assessments yield important insights into real-world settings, providing our study with higher external validity. This study was additionally limited in its assessment of the multicomponent nature of poverty (Duncan and Magnuson, 2003). Here we utilized a cumulative risk framework based on prior research suggesting that the accumulation of risks is more related to poorer outcomes for children than any risk factor alone (e.g., Rutter, 1979; Burchinal et al., 2000, 2006). However, it is possible that specific poverty-related risk factors alone differentially influence academic achievement. Furthermore, our cumulative risk index is limited by its assumption that each poverty-related variable has equal influences on child outcome. Poverty-related risk factors oftentimes co-occur and are highly correlated, and thus it is difficult to tease apart cause-effect relationships to establish mechanisms regarding the multicomponent nature of poverty. Modeling distinct aspects of poverty through the use of animal models has the potential to help disentangle the cause-effect relationships between poverty-related risk factors and child outcomes (Perry et al., 2018). An additional limitation is that we only measured social competence via teacher reports on behavior in school settings. Children have social experiences with peers outside the confines of these settings, which we failed to capture, and thus warrant attention in future research. Similarly, it is possible that teachers' ratings of child prosocial behaviors are specific to the classroom context and biased by perceptions of the child's academic skill-level. Expanding ways in which these constructs are measured in future research would strengthen the present study's findings and interpretations. Lastly, our statistical analysis does not allow for assessment of within person longitudinal changes, which should be the focus of future analyses.

In conclusion, we have provided the first evidence regarding potential autoregressive cross-lagged mediational pathways by which social competence and EF skills may disrupt academic achievement in a high-risk low-income sample. Importantly, we have demonstrated this in real-world contexts and longitudinally across a substantial, ecologically important time in young children's lives. In sum, longitudinal alterations in EF abilities across the early academic years may be one pathway by which poverty-related stressors influence academic abilities. Additionally, altered social competence may further disrupt EF development across formative years of elementary school for at-risk children from low-income families. Such findings suggest that social and EF development may be intrinsically linked, and should always be considered together, which provide important implications. From a preventative perspective, our findings support early screening, and an expansion of early-life screening tools, for both prosocial and EF skills to identify at-risk individuals and foster early academic achievement. Importantly, both EF skills and social skills are malleable and rapidly developing in preschool years, making them feasible targets for preventative interventions to reduce socioecomonic inequality in academic achievement (Howes et al., 1998; Sasser et al., 2017). However, current interventions rarely place equal emphases on targeting EF and social competence together in practice (Bierman et al., 2008; Diamond and Lee, 2011; Christ et al., 2017; Lillard et al., 2017). Our findings promote the importance of integrating existing programs aimed at improving EF or social competence, to optimize the effectiveness of both interventions. In doing so, increasing the prevalence of children in impoverished neighborhoods who are both cognitively and socially ready to succeed in school may prove to be critical to ending lifelong achievement gaps and intergenerational poverty.

## Ethics statement

This study was carried out in accordance with the recommendations of the Institutional Review Board at Pennsylvania State University and the Office of Human Research Ethics at the University of North Carolina with written informed consent from all subjects. All subjects gave written informed consent in accordance with the Declaration of Helsinki.

## Author contributions

RP, SB, and CB contributed to the conception and design of the study. RP performed statistical analyses and wrote the first draft of the manuscript. SB organized the database, performed statistical analyses, and wrote sections of the manuscript. All authors contributed to manuscript revision and read and approved the submitted version.

### Conflict of interest statement

The authors declare that the research was conducted in the absence of any commercial or financial relationships that could be construed as a potential conflict of interest.
